# Neural Dynamic Responses of Monetary and Social Reward Processes in Adolescents

**DOI:** 10.3389/fnhum.2020.00141

**Published:** 2020-04-21

**Authors:** Di Wang, Tongran Liu, Jiannong Shi

**Affiliations:** ^1^CAS Key Laboratory of Behavioral Science, Institute of Psychology, Chinese Academy of Sciences, Beijing, China; ^2^Department of Psychology, University of Chinese Academy of Sciences, Beijing, China

**Keywords:** reward processes, neurodevelopment, adolescence, social reward, monetary reward, event-related potential

## Abstract

Adolescence is an essential developmental period characterized by reward-related processes. The current study investigated the development of monetary and social reward processes in adolescents compared with that in children and adults; furthermore, it assessed whether adolescents had different levels of sensitivity to various types of rewards. Two adapted incentive delay tasks were employed for each participant, and event-related potentials (ERPs) were recorded. The behavioral results showed that both monetary and social rewards could motivate response speed, and participants were more accurate under the monetary reward condition than under the social reward condition. The behavioral performances of individuals increased with age. For the ERP data, the cue-P3, target-P2, target-P3 and feedback-related negativity (FRN) components were investigated to identify reward motivation, emotional arousal, attention allocation and feedback processing. Children and adolescents showed higher motivation (larger cue-P3) to rewards than adults. Adolescents showed larger emotional responses to rewards; that is, they had larger target-P2 amplitudes than adults and shorter target-P2 latencies than children. Children showed stronger emotional reactivity for monetary rewards than for social rewards. All age groups had stronger attentional control (larger target-P3) under the monetary reward condition than under the social reward condition. The present study sheds light on the neurodevelopment of reward processes in children, adolescents and adults and shows that various reward process stages demonstrate different age-related and reward-type-related characteristics.

## Introduction

Adolescence is an essential developmental period characterized by remarkable changes in physical, brain structural domains and hormonal levels, and these changes may further affect adolescents’ functional responsiveness and even behaviors (Steinberg, 2005; Blakemore and Choudhury, 2006; Blakemore, 2008; Casey et al., 2008, 2011; Burnett et al., 2011; Luking et al., 2019; Poon et al., 2019). Rewards can enhance an individual’s motivation, excite positive emotion, optimize the allocation of attention resources and reinforce individual behaviors (Guyer et al., 2006; Delgado, 2007; Bar-Haim et al., 2009; Beck et al., 2010; Helfinstein et al., 2011; Bhanji and Delgado, 2014; Kujawa et al., 2015; Flores et al., 2018). Based on the dual-system theory, the reward system and cognitive control system interact and jointly influence the dynamic changes in reward-related behaviors during individual development (Steinberg, 2008; Galvan, 2010; Shulman et al., 2016; McKewen et al., 2019). Reward system is considered to develop non-linearly (Ernst et al., 2005; Bjork et al., 2010; Somerville and Casey, 2010; Blakemore and Robbins, 2012; Albert et al., 2013; Lamm et al., 2014; Luking, 2015; Ethridge et al., 2017; Kujawa et al., 2018). Adolescents show hypersensitivity to both social and non-social rewards (Martin et al., 2002; Bjork et al., 2004; Cohen et al., 2005; Ernst et al., 2005; Galvan et al., 2006; Casey et al., 2008; Steinberg, 2008; Geier and Luna, 2009; Forbes et al., 2010; Galvan, 2010; Geier et al., 2010; Simon et al., 2010; Somerville et al., 2010; Van Leijenhorst et al., 2010; Wahlstrom et al., 2010; Spear, 2011; Nees et al., 2012; Urošević et al., 2012; Hoogendam et al., 2013; Kennis et al., 2013; Richards et al., 2013; Lamm et al., 2014; Weigard et al., 2014; Braams et al., 2015; Silverman et al., 2015; Foulkes and Blakemore, 2016; Shulman et al., 2016; Steinberg et al., 2017). Rewards can induce intense emotional experiences in adolescents (Ernst et al., 2006; Galvan et al., 2006; Crone and Dahl, 2012; Blakemore and Mills, 2014; Gilbert et al., 2019; van Hoorn et al., 2019), and their motivation toward rewards is an important part of socialization (Kohls et al., 2011; Jia et al., 2013). Abnormal reward process development has been widely seen in children and adolescents with depression (Bress et al., 2015; Luking et al., 2016), autism spectrum disorder (Cox et al., 2015), attention-deficit/hyperactivity disorder (Gonzalez-Gadea et al., 2016), and some other conduct behaviors (Sheffield et al., 2015; Li et al., 2020).

Both monetary and social rewards are widely used in scientific research (Izuma et al., 2008; Knutson et al., 2008; Kohls et al., 2009; Trezza et al., 2011; Forbes and Dahl, 2012; Guyer et al., 2012; Somerville, 2013; Stavropoulos and Carver, 2013; Leotti and Delgado, 2014; Weinberg et al., 2014; Foti et al., 2015; Olino et al., 2015; Anderson, 2016; Foulkes et al., 2017; Cao et al., 2018; Oldham et al., 2018; Altikulaç et al., 2019). Researchers have directly compared behavioral performances and neural responses to social and monetary rewards to investigate whether there were differences between them. Some studies show that monetary and social rewards activate identical neural structures (striatum and medial prefrontal cortex) and comparable scalp topographies and neural response speeds during processes of cue detection, reward anticipation, and feedback evaluation (Izuma et al., 2008; Saxe and Haushofer, 2008; Zink et al., 2008; Guyer et al., 2012; Lin et al., 2012; Olino et al., 2015), which supports the hypothesis of a common neural network (Flores et al., 2015; Oumeziane et al., 2017). However, others suggest that the neural networks for these two reward types are not identical (Spreckelmeyer et al., 2009; Rademacher et al., 2010; Chan et al., 2016), and adult participants are found to be more motivated by monetary rewards than social rewards (Spreckelmeyer et al., 2009; Demurie et al., 2011, 2012; Flores et al., 2015; Wang et al., 2017). The inconsistency of these findings suggests that more research is needed for comparisons between monetary and social reward. In addition, most existing findings were based on adult participants, and the investigation from the developmental perspective might provide more evidences for this topic.

A recent behavioral study explored the developmental differences between monetary and social reward processes in children, adolescents and adults (Wang et al., 2017) by using revised monetary incentive delay (MID) and social incentive delay (SID) tasks (Spreckelmeyer et al., 2009; Rademacher et al., 2010; Kohls et al., 2011; Broyd et al., 2012; Cox et al., 2015; Flores et al., 2015; Wei et al., 2015). It was designed with three different reward magnitudes indicated by the cue (high, low, and non-reward conditions), and the cue was initially presented to promote enhanced anticipatory brain activity that motivated stimulation to prepare for a response (Broyd et al., 2012). Wang et al. (2017) found that both social and monetary rewards could enhance an individual’s response speed, and all age groups showed faster speeds in the high reward magnitude than in the low and non-reward magnitudes. Children and adolescents demonstrated higher motivation for social rewards than for monetary rewards. However, it is still not well known how the neural dynamic differences for social rewards and monetary rewards changed through individual development and how monetary and social rewards motivate the neural responses in adolescents.

According to existing studies using the event-related potential (ERP) technique, the neural dynamic of reward processes further contains the stages of motivation processing, emotional reactivity, cognitive control, and feedback processing (Broyd et al., 2012; Pfabigan et al., 2014, 2015). For motivation processing, the cue-P3 component, a centroparietal positivity that emerges between 300 and 500 ms post cue, relates to the allocation of attention to reward-related cues and reflects the processes of cue detection and motivational approach system activation (Nieuwenhuis et al., 2005; Goldstein et al., 2006; Kohls et al., 2011; Broyd et al., 2012; Cox et al., 2015; Flores et al., 2015; Wei et al., 2015; Oumeziane et al., 2017). It has been indicated that higher reward magnitudes or more desirable rewards induced larger cue-P3 amplitudes compared to the cue-P3 amplitudes induced by lower reward magnitudes or less desirable rewards (Schacht and Sommer, 2009; Broyd et al., 2012; Pfabigan et al., 2014). With regard to emotional reactivity processing, target-P2, which is a positive peak at the anterior and central electrode sites approximately 200 ms following the target, is amplified by valuating the emotion of the reward stimuli (Kaltwasser et al., 2013; Wei et al., 2015). For the stage of cognitive control processing, target-P3 reflects the attentional capture of a reward and the cognitive control process (Broyd et al., 2012; Flores et al., 2015; Wei et al., 2015). During the feedback stage, the feedback-related negativity (FRN) over frontal and central brain areas peaking between 200 and 400 ms reflects the cognitive monitoring on different feedback; the more an individual relies on external feedback, the larger the FRN amplitude (Eppinger et al., 2009; Hämmerer et al., 2011; Crowley et al., 2013). FRN is typically regarded to be low for the reward gain condition and high for the reward loss condition (Holroyd and Coles, 2002; Holroyd et al., 2003; Heldmann et al., 2008). Developmental studies have shown that children have enhanced FRN in response to feedback compared to that of adolescents and adults, but less differentiation between positive and negative feedback (Hämmerer et al., 2011; Crowley et al., 2013; Ferdinand and Kray, 2014), which might indicate that children react strongly to external feedback. However, the age-related neural processing differences between monetary and social rewards during reward-related motivation, emotional sensitivity and attentional allocation are still unclear.

The aim of the current study was to investigate the development of neural mechanisms for monetary and social reward processing in adolescents compared with children and adults. The period of early adolescence is essential for the development of reward processes and emotional abilities (Crone and Dahl, 2012; Albert et al., 2013; Blakemore and Mills, 2014). We chose a group of 8 years old children as a comparative group, that was because the understanding of the concept of money was fully established by age 8 (Grunberg and Anthony, 1980; Berti and Bombi, 1981). Our first hypothesis was that adolescents may have higher motivation (larger cue-P3 responses) toward social rewards than toward monetary rewards. Second, it was further hypothesized that adolescents may show stronger emotional activities (larger target-P2) toward social rewards than children and adults. Third, we hypothesized that high rewards would induce stronger neural responses than low rewards.

## Materials and Methods

### Participants

Individuals from three age groups participated in this experiment. Data from 30 children (15 males; 8.1–9.0 years old, mean age of 8.6 years), 30 adolescents (15 males; 12.2–14.5 years old, mean age of 13.5 years), and 30 adults (15 males; 23.3–26.7 years old, mean age of 24.8 years) were analyzed. The adults were undergraduate and graduate students at a university. The children and adolescents were recruited from a local primary school and a local middle school. All participants were right-handed with normal or corrected-to-normal vision and were free from neurological or psychiatric disorders. The parents of the participants who were children and adolescents provided written informed consent before these individuals participated in the present study, while adult participants provided their own written consent. The present study was approved by the Ethics Committee of the Institute of Psychology, Chinese Academy of Sciences, and it was carried out in accordance with the Code of Ethics of the World Medical Association (Declaration of Helsinki).

### Materials and Procedures

**FIGURE 1:**
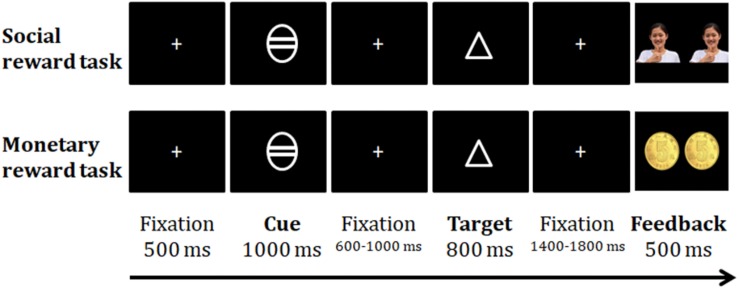
Examples of the trial sequence in monetary and social tasks.

All data were collected in a dimly lit and shielded room. The presentation of stimuli and the recording of error rates and reaction times (RTs) were controlled by E-Prime 2.0 software (Psychology Software Tools, Pittsburgh, PA, United States). There were three stages of the study: the baseline RT measurement, ERP experiment, and subjective rating (Figure 1). The current study was designed based on a previous behavioral study with several refinements (Wang et al., 2017). During the baseline RT measurement stage, participants were blinded to the experimental purpose and design. After completing the baseline RT measurements but before the ERP experiment, the participants were explicitly told that the ERP experiment was about rewards, and they were informed of the ERP experimental procedures, rules and incentive standards for the monetary and social rewards. All participants clearly knew the connection between their performance and the final rewards (real money and a paper honor certificate) before the ERP tasks. For the monetary reward setup, participants were told that a picture of one or two coins would be presented to indicate their good performance during each trial, and at the end of the money task, 9-45 real Chinese Yuan would be issued to the qualifying participants. For the social reward setup, participants were told that they would receive a praise from a picture of a real experimenter on the screen at the end of each trial, and a paper honor certificate would be given to the qualifying participants after completing the task. The certificate was based on each participant’s performance in the ERP task compared to his/her performance in the baseline stage. The honor certificates displayed different sentences as follows: “Congratulations for receiving XXX social approval points (ranging from 91 to 180 points), and you received the XXX (first-fifth) place prize in the psychological experiment!” There was no difference in the exterior of the honor certificates from fifth prize to first prize.

#### Baseline RT Measurements

Participants were instructed to respond to the shape of a target figure (visual angle of the square stimulus: 2.12° × 2.12°, visual angle of the triangle stimulus: 3.22° × 2.79°) by pressing the left or right button of a computer mouse. A white fixation cross (0.48° × 0.48° visual angle) appeared at the center of a black screen for 600–1000 ms at the beginning of each trial. The target figure was subsequently presented in the center of the screen for 800 ms. The participants were instructed to respond to the target shape as quickly and accurately as possible. Then, the fixation cross was presented for 1400–1800 ms, followed by the feedback stimulus for 500 ms (“√”: 3.99° × 2.64° for a correct response, or “ × ”: 2.64° × 2.64° for an incorrect response). There were 40 trials in this stage to allow participants to become familiar with the task and to ensure that they all understood the experimental instructions. Notably, the average RT of correct responses for each participant was used as his or her baseline RT in the next stage.

#### ERP Experiment

Both social and monetary tasks consisted of 180 trials in three continuous blocks. The reward magnitudes for high rewards, low rewards and non-rewards accounted for one-third of each block and were randomly presented. Each trial began with a white fixation cross (0.4° × 0.4°) at the center of a black screen for 500 ms, followed by a cue for 1000 ms (2.07° × 2.07°). The magnitude of the potential reward was indicated by the number of horizontal lines in the cue. A white circle with 2 lines indicated the high reward condition, whereas an empty white circle or a circle with 1 line indicated the non-reward or low reward condition, respectively. The cue-target interval was 600–1000 ms, and the target figure (square or triangle) was presented for 800 ms in the center of the screen. The association between the response buttons and the shapes was consistent with that in the baseline RT measurement stage. Participants were instructed to respond to the shape of the target as quickly and accurately as possible. The assignments of the response buttons to the target figures and the sequence of the social and monetary reward tasks were counterbalanced across the participants in each age group.

After the target figure was displayed, the fixation cross was presented again for 1400–1800 ms, followed by a feedback stimulus for 500 ms. For the non-reward trials, a “√” or “ × ” symbol was displayed as the feedback stimulus according to whether the response was correct or not. In the low reward trials, a photo of a Chinese female (26.8 years old) (3.88° × 3.88°) with a happy expression making a thumbs-up gesture or one 0.5 Chinese Yuan coin (3.88° × 3.88°) was presented as the social or monetary low-reward feedback stimulus if the participant provided accurate responses that were faster than their baseline RT. In the high reward condition, two 0.5 Chinese Yuan coins (8.01° × 3.88°) or two identical happy faces of the young woman were presented as feedback. To quantify low and high rewards, 1 and 2 points, respectively, were added to the total points for the corresponding reward type. The total points equaled the cumulative number of coins or smiling faces the participants had received in the corresponding tasks. For the low and high reward trials, if participants responded incorrectly, they would see an ” × ” symbol without any additional points and rewards; if participants responded correctly but with a longer RT than that in the baseline measurement, they would see a ”√” symbol without no additional points or rewards. The fixation point was subsequently presented for 1100–1600 ms. The cumulative points were reported at the end of each block to ensure that participants were aware of their performance in the task. After completing the task, the total points (i.e., the sum of the coin values or smiling faces) that the participants had earned for that reward type were presented. The total number of points for each task was 180, and after the tasks, only participants with a total score above 90 qualified for the monetary and social reward. Different scores corresponding to different reward levels are shown in Table 1.

**TABLE 1:** Reward standard in monetary and social reward tasks.

**Total points**	**Monetary reward task**	**Social reward task**
0–90 points	0 Chinese Yuan	0
91–108 points	9 Chinese Yuan	Fifth prize
109–126 points	18 Chinese Yuan	Fourth prize
127–144 points	27 Chinese Yuan	Third prize
145–162 points	36 Chinese Yuan	Second prize
163–180 points	45 Chinese Yuan	First prize

#### Subjective Rating

Subjective ratings were conducted after participants completed the ERP experiment, and they were instructed to rate their general motivation for monetary and social rewards using 7-point Likert scales that ranged from 1 (do not want it at all) to 7 (want it very much). There was no further subjective rating for the cues.

### ERP Recordings

ERP recordings were obtained from 40 scalp sites using Ag/AgCl electrodes embedded in an elastic cap at locations from the extended International 10-20 system (Neuroscan; Compumedics, EI Paso, TX, United States). These electrodes were referenced to the A1 electrode, which was located at the left mastoid during recording and referenced to the average of the right (A2) and left mastoid (A1) potentials offline. Horizontal and vertical electrooculograms (EOGs) were recorded by two electrodes on the outer canthi of both eyes and two electrodes on the inferior and superior areas of the left eye, respectively. Impedance was kept below 5 kΩ, and electroencephalograph signals were on-line filtered with a bandpass of 0.1–100 Hz and sampled at a rate of 1000 Hz. Each averaging epoch lasted 1000 ms, with an additional 100 ms prior to the stimulus onset to enable baseline correction. Erroneous trials were excluded from the analyses. Trials contaminated by eye blink artifacts or body movements and those with voltages that exceeded ± 100 μV relative to the 100 ms baseline at any electrode were excluded from further analyses. The averaged ERPs were further filtered off-line with bandwidths of 0.1–30 Hz (slope: 24 dB/octave; zero phase shift).

### Data Analysis

Based on previous ERP studies on reward processing and a visual inspection of the current data, we calculated post-cue responses over the central and parietal electrodes (average of C3, Cz, C4, CP3, CPz, and CP4), indexing the cue-P3 component with a time window of 400–600 ms (Broyd et al., 2012). We calculated the target-P2 component over the frontal and central electrodes (average of F3, Fz, F4, FC3, FCz, FC4, C3, Cz, and C4) with a time window of 150-280 ms and the target-P3 component over the central and parietal electrodes (average of C3, Cz, C4, CP3, CPz, and CP4) for the interval 280–600 ms after the stimulus onset (Wei et al., 2015). In addition, the FRN component was calculated for the frontal and central midline electrodes (average of Fz, FCz, and Cz) during the 200–400 ms interval after the stimulus-onset (Crowley et al., 2013).

The behavioral performance (mean RT and error rate), mean amplitudes and peak latencies of ERP components (cue-P3, target-P2, target-P3, and FRN) were analyzed via analyses of variance (ANOVAs) with two within-subject factors, Reward Type (monetary, social) and Reward Magnitude (non-reward, low reward, high reward), and two between-subject factors, Age group (children, adolescents, adults) and Gender (male, female). For subjective ratings, the Reward type was the within-participant factor, and the Age group and Gender were the between-subject factors. For the ANOVA analyses, we mainly reported the age-related analyses on the following main effects and interaction effects: the main effects of Age group and Reward type, and the interactions of Age group × Reward type, Age group × Reward magnitude, Age group × Reward type × Reward magnitude. All repeated-measures ANOVAs were corrected with Greenhouse–Geisser corrections, and the Bonferroni tests were used to conduct *post hoc* comparisons. The hypothesis-related findings are presented in the text. For the main effects of Age group, the *post hoc* tests were on the comparisons of children vs. adolescents vs. adults (number of 3 tests for Bonferroni correction, *p* < 0.05/3 = 0.017). For the main effects of Reward type, the *post hoc* tests were on the comparisons of monetary rewards vs. social rewards (number of 1 test for Bonferroni correction, *p* < 0.05). For the main effects of Reward magnitude, the *post hoc* tests were on the comparisons of high magnitude vs. low magnitude vs. non-reward (number of 3 tests for Bonferroni correction, *p* < 0.05/3 = 0.017). For the interaction between Age group × Reward type, the *post hoc* tests were on the comparisons of children vs. adolescents vs. adults under each reward type level (monetary reward, social reward) (number of 6 tests for Bonferroni correction, *p* < 0.05/6 = 0.008), and also the comparisons of monetary rewards vs. social rewards under each age group level (number of 3 tests for Bonferroni correction, *p* < 0.05/3 = 0.017). For the interaction between Age group × Reward magnitude, the *post hoc* tests were on the comparisons of children vs. adolescents vs. adults under each magnitude level (high reward, low reward, non-reward) (number of 9 tests for Bonferroni correction, *p* < 0.05/9 = 0.006). For the interaction between Age group × Reward type × Reward magnitude, the *post hoc* tests were on the comparisons of children vs. adolescents vs. adults under each magnitude level and each type reward (number of 18 tests for Bonferroni correction, *p* < 0.05/18 = 0.003), and *post hoc* tests were the comparisons of monetary rewards vs. social rewards under each age group level and under each magnitude level (number of 9 tests for Bonferroni correction, *p* < 0.05/9 = 0.006). Correlation analyses between behavioral data (RT and error rates) and ERP data (amplitudes and latencies of cue-P3, target-P2 and target-P3) were also conducted.

## Results

**FIGURE 2:**
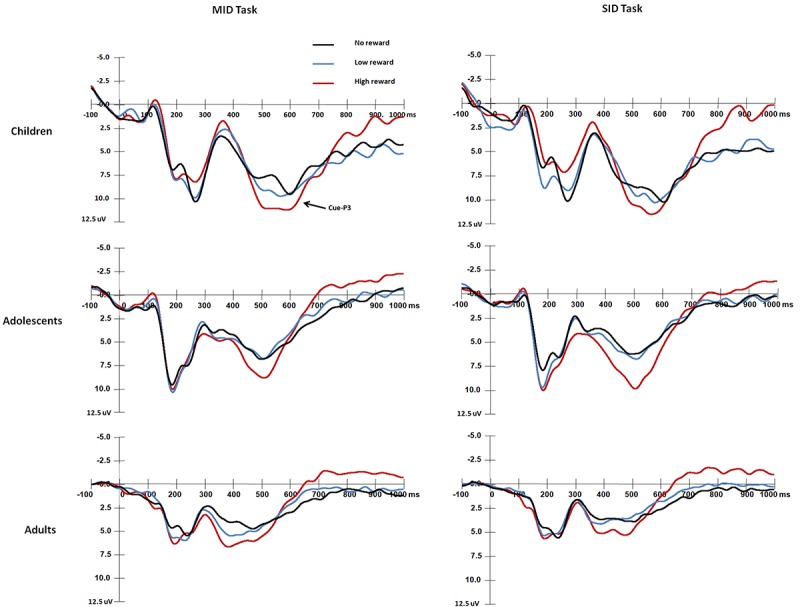
Grand average waveforms of cue-P3 components over central-parietal areas (CPz) in response to the presentation of the cue stimuli for each age group.

**FIGURE 3:**
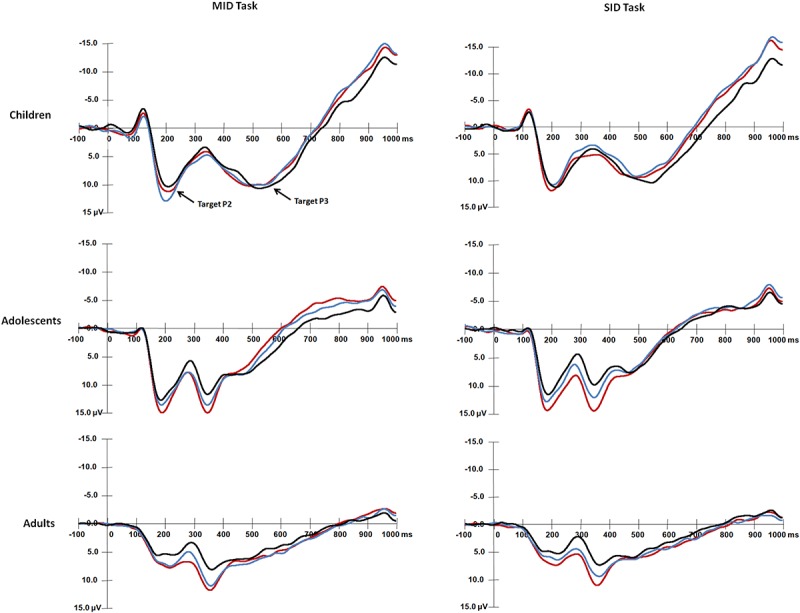
Grand average waveforms of target-P2 and target-P3 components over central areas (Cz) in response to the presentation of the target stimuli for each age group.

**FIGURE 4:**
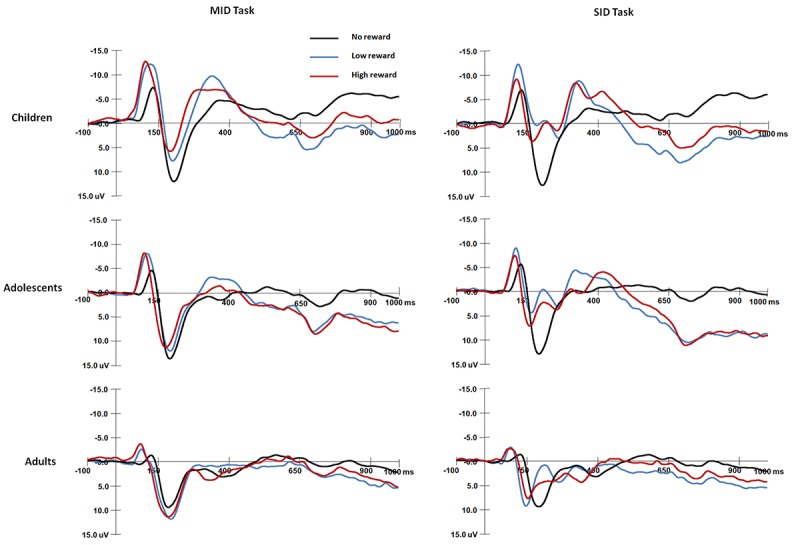
Grand average waveforms of FRN components over fronto-central areas (FCz) in response to the presentation of the feedback stimuli for each age group.

The mean RTs and response error rates in both the baseline stage and ERP tasks and the mean subjective rating scores for both monetary and social rewards are presented in Table 2. The mean amplitude (μV) and peak latency (ms) for each ERP component with valid trial numbers in all conditions are presented in Table 3. Both significant and non-significant results of ANOVA analyses for the behavioral and ERP data are displayed in Table 4. The grand average waveforms of cue-P3 are presented in Figure 2, those for target-P2 and target-P3 are shown in Figure 3, and those for FRN are displayed in Figure 4.

**TABLE 2:** Mean and standard deviation of reaction time (ms) and error rates in the baseline stage and ERP tasks, and subjective ratings for both rewards.

**Age group**	**Behavioral data**	**Baseline stage**	**Monetary reward task**			**Social reward task**		
			**Non- reward**	**Low reward**	**High reward**	**Non- reward**	**Low reward**	**High reward**
Children	Reaction Time	646 (98)	614 (101)	578 (88)	565 (89)	610 (89)	581 (94)	563 (95)
	Error rates	4.23 (3.77)	7.83 (6.24)	6.10 (4.51)	8.21 (6.04)	7.85 (5.80)	7.33 (5.64)	7.31 (5.56)
	Subjective rating	N/A	4.87 (1.57)			5.77 (1.38)		
Adolescent	Reaction Time	471 (59)	457 (54)	420 (42)	406 (47)	462 (55)	426 (41)	414 (41)
	Error rates	4.58 (3.48)	4.83 (4.18)	5.33 (5.33)	5.33 (4.54)	6.17 (5.40)	6.50 (5.40)	5.00 (3.94)
	Subjective rating	N/A	4.50 (0.86)			5.10 (1.16)		
Adult	Reaction Time	508 (52)	471 (59)	425 (38)	408 (35)	465 (53)	426 (36)	415 (37)
	Error rates	2.35 (2.5)	2.06 (2.38)	2.08 (1.89)	1.88 (2.43)	2.88 (2.87)	2.82 (4.02)	1.58 (1.99)
	Subjective rating	N/A	5.33 (1.06)			5.37 (1.33)		

**TABLE 3:** Mean and Standard Deviation of valid trial numbers, mean amplitude (μV) and peak latency (ms) for each age group.

**Age group**	**ERP data**		**Monetary reward task**			**Social reward task**		
			**Non- reward**	**Low reward**	**High reward**	**Non- reward**	**Low reward**	**High reward**
Children	Cue-P3	Trial numbers	48.3 (8.2)	48.9 (8.1)	48.5 (8.6)	46.1 (7.3)	47.5 (6.3)	46.1 (8.1)
		Amplitude	9.5 (4.7)	10 (4.4)	10.6 (4.9)	9.9 (5.1)	10 (5.5)	11.1 (6.3)
		Latency	517 (45)	518.1 (39.4)	518.8 (34)	507 (45.6)	512.9 (42.5)	514.3 (41.5)
	Target-P2	Trial numbers	50.2 (7.4)	50.2 (7.7)	49.1 (7.7)	48.3 (6.3)	49.3 (5.7)	48.1 (6.3)
		Amplitude	12.7 (4.05)	15.0 (4.7)	13.7 (4.1)	13.4 (4.6)	12.6 (4.6)	13.7 (3.7)
		Latency	217.7 (21.3)	211.6 (21.9)	215.4 (24.1)	219.3 (19.4)	215.9 (23.2)	212.3 (22.2)
	Target-P3	Amplitude	13.8 (5.3)	15.1 (5.6)	15.5 (5.7)	14.1 (5.3)	13.58 (4.54)	15.47 (5.08)
		Latency	395.8 (76.2)	382.6 (67.3)	390.1 (68.5)	383.8 (67.1)	388.2 (67.1)	383.0 (68.6)
	FRN	Trial numbers	74.6 (20.2)	44.1 (11.3)	46.1 (12.1)	70.1 (15.6)	43.6 (9.5)	45.1 (8.3)
		Amplitude	−6.3(4.7)	−12.3(6.2)	−10.7(5.5)	−5.1(3.4)	−10.9(7.1)	−12.2(5.0)
		Latency	341.6 (42.8)	329 (33.9)	300.8 (36.3)	342.3 (36.2)	329.9 (37.7)	331.0 (45.8)
Adolescents	Cue-P3	Trial numbers	50.3 (6.7)	49.6 (7.3)	49.7 (6.8)	46.7 (7.1)	46.4 (6.4)	47.4 (5.8)
		Amplitude	7.7 (4.6)	7.4 (3.5)	9.1 (4.3)	7.5 (4)	7.8 (4.2)	9.4 (4.9)
		Latency	494.3 (44.6)	481.2 (48.4)	491.7 (40.1)	502.6 (51.3)	492.2 (42.8)	492 (39)
	Target-P2	Trial numbers	51.3 (7.1)	51.1 (6.8)	51.4 (6.4)	48.8 (6.1)	48.9 (6.1)	47.9 (6.1)
		Amplitude	12.3 (4.1)	13.2 (4.7)	14.0 (4.9)	11.2 (4.8)	12.5 (4.2)	13.7 (4.2)
		Latency	198.7 (22.8)	203.0 (22.2)	200.1 (21.4)	198.7 (22.7)	198.5 (22.6)	199.7 (23.5)
	Target-P3	Amplitude	13.3 (4.7)	15.0 (5.3)	15.7 (5.7)	11.6 (5.1)	13.3 (5.4)	15.6 (5.8)
		Latency	376.4 (46.8)	359.6 (37.7)	362.9 (41.9)	383.4 (44.1)	368.2 (41.2)	365.8 (40.7)
	FRN	Trial numbers	73.5 (19.7)	41.6 (8.5)	44.1 (8.3)	71.3 (18.4)	40.5 (9.1)	42.6 (8.2)
		Amplitude	−1.0(3.4)	−5.4(5.5)	−4.6(4.4)	−2.0(4.1)	−7.1(5.5)	−5.5(5.4)
		Latency	312.1 (28.5)	336.5 (40.7)	314.2 (53.1)	317.5 (34.2)	305.4 (51.4)	324.8 (70.5)
Adults	Cue-P3	Trial numbers	53.7 (6.3)	54.6 (4.1)	53.7 (5.4)	53.4 (7.5)	53.8 (6.4)	54.4 (6.9)
		Amplitude	5.5 (3.2)	5.9 (2.8)	7 (2.9)	4.4 (2.7)	4.6 (3.4)	5.9 (3.3)
		Latency	473.2 (47.8)	451.3 (43.3)	449.1 (45.6)	468 (45)	456.9 (53.1)	458.7 (43.1)
	Target-P2	Trial numbers	54.2 (6.1)	55.6 (4.7)	54.3 (6.2)	53.5 (6.8)	54.5 (6.2)	54.5 (6.8)
		Amplitude	7.7 (2.6)	8.8 (3.0)	9.2 (2.7)	7.2 (2.8)	8.1 (2.8)	8.9 (2.8)
		Latency	203.9 (31.5)	210.1 (30.2)	212.7 (32.9)	200.2 (27.8)	202.1 (33.8)	203.2 (26.5)
	Target-P3	Amplitude	9.2 (3.3)	11.1 (3.2)	11.7 (3.7)	8.6 (3.1)	9.8 (3.6)	11.0 (3.6)
		Latency	389.5 (55.5)	375.5 (40.1)	362.3 (41.6)	383.6 (54.3)	375.4 (47.7)	368.7 (42.1)
	FRN	Trial numbers	67.4 (13.2)	48.6 (10.9)	52.2 (9.3)	66.9 (12.7)	48.9 (8.3)	51.4 (7.6)
		Amplitude	−0.5(3.5)	−1.6(4.6)	−1.0(4.5)	−0.7(3.2)	−2.5(4.1)	−1.3(3.2)
		Latency	300.3 (38.1)	324.1 (43.4)	285.6 (43.2)	306.4 (29.0)	269.5 (70.5)	309.8 (63.5)

**TABLE 4:** ANOVA results of behavioral data and ERP latencies and amplitudes.

	**Reaction time**		**Error** **rate**		**Cue-P3 latency**		**Cue-P3 amplitude**		**Target-P2 latency**		**Target-P2 amplitude**		**Target-P3 latency**		**Target-P3 amplitude**		**FRN** **latency**		**FRN amplitude**	
	***F***	***p***	***F***	***p***	***F***	***p***	***F***	***p***	***F***	***p***	***F***	***p***	***F***	***p***	***F***	***p***	***F***	***p***	***F***	***p***
A	68.07	<0.001	15.28	<0.001	19.3	<0.001	12.76	<0.001	3.63	0.03	21.91	<0.001	1.04	0.36	10.01	<0.001	8.46	<0.001	42.07	<0.001
T	0.22	0.64	2.6	0.11	0.16	0.69	0.47	0.50	3.40	0.07	4.85	0.03	0.07	0.80	7.58	0.007	0.05	0.82	1.88	0.17
M	140.82	<0.001	0.7	0.45	3.73	0.03	18.72	<0.001	0.21	0.81	23.21	<0.001	13.03	<0.001	49.44	<0.001	2.31	0.10	68.87	<0.001
A × T	0.27	0.76	0.4	0.64	2.01	0.14	2.60	0.08	2.91	0.06	0.08	0.93	1.64	0.20	0.59	0.56	2.51	0.09	1.96	0.15
A × M	0.44	0.71	2.1	0.09	2.54	0.04	0.45	0.75	3.63	0.007	1.94	0.11	2.28	0.07	2.50	0.048	2.51	0.04	10.39	<0.001
M × T	1.57	0.22	3.5	0.03	0.49	0.61	0.19	0.82	0.94	0.39	3.31	0.04	1.12	0.33	3.88	0.025	19.86	<0.001	1.02	0.36
A × M × T	0.77	0.54	0.3	0.87	0.60	0.66	0.26	0.90	0.91	0.46	3.23	0.01	0.82	0.51	1.30	0.28	3.00	0.02	1.78	0.14

*A, age group; T, reward type; M, reward magnitude.*

### Behavioral Results

#### Reaction Time

The results indicated a main effect of Age group, *F*(2,87) = 68.06, *p* < 0.001, η*^2^* = 0.61; the *post hoc* comparisons indicated significantly faster RTs for adults [*t*(58) = −9.97, *p* < 0.001] and adolescents [*t*(58) = −10.23, *p* < 0.001] than for children. The difference between adolescents and adults did not reach significance, *t*(58) = −0.26, *p* = 1.00. In addition, a main effect of Reward magnitude was identified, *F*(2,174) = 140.82, *p* < 0.001, η*^2^* = 0.618; *post hoc* comparisons indicated significantly faster RTs in the low [*t*(89) = −11.71, *p* < 0.001] and high reward conditions [*t*(89) = −12.91, *p* < 0.001] than in the non-reward condition, and a faster RT in the high reward condition than in the low reward condition, *t*(89) = −6.97, *p* < 0.001. The other main effects and interaction effects were not significant (*ps* > 0.05).

#### Error Rate

The results showed a main effect of Age group, *F*(2,87) = 15.28, *p* < 0.001, η*^2^* = 0.26, and the error rate was lower for adults than for children [*t*(58) = −5.20, *p* < 0.001] and adolescents [*t*(58) = −3.30, *p* < 0.005]. The difference between children and adolescents was not significant, *t*(58) = 1.90, *p* = 0.15. The other main effects and interaction effects were not significant (*ps* > 0.05).

#### Subjective Rating

The Reward type factor significantly interacted with the Age group, *F*(2,87) = 3.14, *p* < 0.05, η*^2^* = 0.067. *Post hoc* comparisons indicated that children [*t*(29) = 3.63, *p* < 0.001] and adolescents [*t*(29) = 2.42, *p* = 0.018 < 0.05] rated social rewards significantly higher than monetary rewards. For adults, the subjective rating difference between monetary and social rewards was not significant, *t*(29) = −0.13, *p* = 0.89. The other main effects and interaction effects were not significant (*ps* > 0.05).

### ERP Results

#### Cue-P3 Amplitude

With regard to the cue-P3 amplitude (Figure 2), the results showed a main effect of Age group, *F*(2,87) = 12.76, *p* < 0.001, η*^2^* = 0.23, and adults exhibited smaller cue-P3 amplitudes than children [*t*(58) = −4.99, *p* < 0.001] and adolescents [*t*(58) = −2.83, *p* = 0.006 < 0.017]. There were no differences between children and adolescents [*t*(58) = 2.21, *p* = 0.03 > 0.017]. In addition, a main effect of Reward magnitude was identified, *F*(2,174) = 18.72, *p* < 0.001, η*^2^* = 0.18, and the cue-P3 amplitude was larger in the high reward condition than that in the low and non-reward conditions (*ps* < 0.001). The other main effects and interaction effects were not significant (*ps* > 0.05).

#### Cue-P3 Latency

For the cue-P3 latency, the main effect of Age group was significant [*F*(2,87) = 19.31, *p* < 0.001, η*^2^* = 0.31], and adults had a shorter cue-P3 latency than children and adolescents (*ps* < 0.001). There were no differences between children and adolescents (*p* > 0.05). The main effect of Reward magnitude was also significant, *F*(2,87) = 3.73, *p* < 0.05, η*^2^* = 0.04, that was, the cue-P3 latency was shorter under low reward condition than that under no reward condition [*t*(89) = 2.57, *p* = 0.012 < 0.017]. There were no differences between high and no reward conditions in cue-P3 latencies (*p* > 0.05). The other main effects and interaction effects were not significant (*ps* > 0.05).

#### Target-P2 Amplitude

For the mean amplitude of target-P2 (Figure 3), the main effect of Age group was significant, *F*(2,87) = 21.91, *p* < 0.001, η*^2^* = 0.34, and adults had smaller target-P2 amplitudes than children [*t*(58) = 6.05, *p* < 0.001] and adolescents [*t*(58) = 5.28, *p* < 0.001]. There were no differences between children and adolescents (*p* > 0.05). The main effect of Reward type was significant [*F*(1,87) = 4.85, *p* < 0.05, η*^2^* = 0.05], and that for target-P2 was larger for monetary rewards than for social rewards. The main effect of Reward magnitude was significant, *F*(2,174) = 23.21, *p* < 0.001, η*^2^* = 0.21, and target-P2 was smaller in the non-reward condition than in the high [*t*(89) = 6.33, *p* < 0.001] and low reward conditions [*t*(89) = 4.46, *p* < 0.001].

The interaction among Age group, Reward type and Reward magnitude was significant, *F*(4,174) = 3.23, *p* = 0.014, η*^2^* = 0.07. Adults had smaller target-P2 amplitudes than children and adolescents (*ps* < 0.001). For monetary rewards, children had larger target-P2 values in the low reward condition than in the non-reward condition [*t*(29) = 3.82, *p* < 0.001]. For social rewards, adolescents had smaller target-P2 amplitudes in the non-reward condition than in the high [*t*(29) = 4.85, *p* < 0.001] and low conditions [*t*(29) = 3.05, *p* = 0.009]. Adults had larger target-P2 amplitudes in the high condition than in the non-reward condition [*t*(29) = 3.44, *p* = 0.003 < 0.005]. Moreover, children had larger target-P2 amplitudes for money rewards than for social rewards in the low reward condition [*t*(29) = 3.26, *p* = 0.002 < 0.005]. The other main effects and interaction effects were not significant (*ps* > 0.05).

#### Target-P2 Latency

For target-P2 latency, the main effect of Age group was significant, *F*(2,87) = 3.63, *p* < 0.05, η*^2^* = 0.08, and adolescents had a shorter target-P2 latency than children [*t*(58) = 2.66, *p* = 0.027 > 0.017]. There were no differences between children and adults (*p* > 0.05).

The interaction between Reward magnitude and Age group was significant, *F*(4,174) = 3.63, *p* < 0.01, η*^2^* = 0.08, and adolescents had shorter target-P2 latency than children in the non-reward condition [*t*(29) = 3.29, *p* = 0.004 < 0.006]. The other main effects and interaction effects were not significant (*ps* > 0.05).

#### Target-P3 Amplitude

For the target-P3 mean amplitude (Figure 3), the main effect of Age group was significant [*F*(2,87) = 10.01, *p* < 0.001, η*^2^* = 0.19], and children and adolescents had larger target-P3 amplitudes than adults [children vs. adults: *t*(58) = 4.06, *p* < 0.001; adolescents vs. adults: *t*(58) = 3.62, *p* < 0.001]. The main effect of Reward type was significant [*F*(1,87) = 7.58, *p* < 0.01, η*^2^* = 0.08], and that for target P3 was larger for monetary rewards than for social rewards. The significant main effect of Reward magnitude was significant, *F*(2,174) = 49.44, *p* < 0.001, η*^2^* = 0.36; the target-P3 amplitude was larger in the high reward condition than in the low and non-reward conditions [high vs. low: *t*(89) = 5.59, *p* < 0.001; high vs. non: t(89) = 9.03, *p* < 0.001], and the target-P3 amplitude was larger in the low reward condition than in the non-reward condition [*t*(89) = 5, *p* < 0.001].

The interaction between Reward magnitude and Age group was significant, *F*(4,174) = 2.50, *p* < 0.05, η*^2^* = 0.05. Children and adolescents had larger target-P3 amplitudes than adults (*ps* < 0.001). For children, target-P3 amplitudes in the high reward condition were larger than those in the non-reward conditions [high vs. non: *t*(29) = 3.28, *p* < 0.006]. For adolescents, target-P3 amplitudes in the high reward condition were larger than those in the low and non-reward conditions [high vs. low: *t*(29) = 4.02, *p* < 0.001; high vs. non: *t*(29) = 6.96, *p* < 0.001]; target-P3 amplitudes in the low reward condition were larger than those in the non-reward condition [*t*(29) = 4.11, *p* < 0.001]. For adults, target-P3 amplitudes in the non-reward condition were smaller than those in the high and low conditions [high vs. non: *t*(29) = 5.43, *p* < 0.001; low vs. non: *t*(29) = 3.77, *p* < 0.001]. The other main effects and interaction effects were not significant (*ps* > 0.05).

The interaction between Reward type and Reward magnitude was significant, *F*(2,174) = 3.88, *p* < 0.05, η*^2^* = 0.043. Only in the low reward condition, target-P3 amplitudes were larger for monetary rewards than for social rewards [*t*(89) = 4.03, *p* < 0.001]. In addition, for monetary rewards, target-P3 amplitudes in the non-reward condition were smaller than those in the high and low conditions [high vs. non: *t*(89) = 6.31, *p* < 0.001; low vs. non: *t*(89) = 4.91, *p* < 0.001]. For social rewards, target-P3 amplitudes in the high reward condition were larger than those in the low and non-reward conditions [high vs. low: *t*(89) = 6.26, *p* < 0.001; high vs. non: *t*(89) = 6.82, *p* < 0.001].

#### Target-P3 Latency

For target-P3 latency, the main effect of Reward magnitude was identified, *F*(2,174) = 13.03, *p* < 0.001, η*^2^* = 0.13, and target-P3 latency was longer in the non-reward condition than in the high [*t*(89) = 4.43, *p* < 0.001] and low reward conditions [*t*(89) = 3.64, *p* < 0.001]. The other main effects and interaction effects were not significant (*ps* > 0.05).

#### Feedback-Related Negativity (FRN) Amplitude

For the FRN amplitude (Figure 4), the main effect of Age group was significant, *F*(2,87) = 42.1, *p* < 0.001, η*^2^* = 0.49; children had more negative FRN amplitudes than adolescents [*t*(58) = −5.77, *p* < 0.001], and adolescents had more negative FRN amplitudes than adults [*t*(58) = −3.29, *p* = 0.004]. The main effect of Reward magnitude was significant, *F*(2,174) = 68.9, *p* < 0.001, η*^2^* = 0.44, and FRN was less negative in the non-reward condition than in the high [*t*(89) = 9.32, *p* < 0.001] and low conditions [*t*(89) = 1.01, *p* < 0.001].

The interaction between Reward magnitude and Age group was significant, *F*(4,174) = 10.4, *p* < 0.001, η*^2^* = 0.19; for children and adolescents, FRN amplitudes were more negative in the high and low reward conditions than in the non-reward condition (*ps* < 0.001); however, adults had comparable FRN amplitudes in the reward and non-reward conditions (*ps* > 0.05). The other main effects and interaction effects were not significant (*ps* > 0.05).

#### Feedback-Related Negativity (FRN) Latency

For FRN latency, the main effect of Age group was significant, *F*(2,87) = 8.46, *p* < 0.001, η*^2^* = 0.16; adults had shorter FRN latencies than children [*t*(58) = −4.06, *p* < 0.001], but there were no differences between adults and adolescents [*t*(58) = −2.61, *p* = 0.032 > 0.017].

The interaction among Reward type, Reward magnitude and Age group was significant [*F*(4,174) = 3, *p* < 0.05, η*^2^* = 0.07]. For monetary rewards, children had longer FRN latencies than adolescents [*t*(58) = 3.09, *p* < 0.008] and adults [*t*(58) = 4.33, *p* < 0.001] in the non-reward condition. For social rewards, adults had shorter FRN latencies than children [*t*(58) = 4.26, *p* < 0.001]; children had longer FRN latencies than adults [*t*(58) = 4.19, *p* < 0.001] in the non-reward condition. For adolescents and adults, FRN latencies were shorter for social rewards than monetary rewards in the low reward condition [adolescent: *t*(29) = 3.05, *p* < 0.005; adult: *t*(29) = 5.34, *p* < 0.001]; For monetary rewards, children had shorter FRN latencies in the high reward condition than in the low [*t*(29) = 3.22, *p* < 0.005] and non-reward conditions [*t*(29) = 4.9, *p* < 0.001]; For money rewards, adults had longer FRN latencies in the low reward condition than in the high [*t*(29) = 4.4, *p* < 0.001]; for social rewards, adults had shorter FRN latencies in the low reward condition than in the high [*t*(29) = 3.63, *p* < 0.001] and non-reward conditions [*t*(29) = 3.43, *p* < 0.005]. The other main effects and interaction effects were not significant (*ps* > 0.05).

### The Correlation Between Behavioral and ERP Data

With regard to each age group, the correlation between behavioral data and ERP data was strictly corrected by *p* = 0.05/10 = 0.005 for each experimental condition because there were 10 independent factors in the correlation matrix (RT, error rates, amplitudes and latencies of cue-P3, target-P2, target-P3 and FRN). For Adolescents, RTs in high social reward condition positively correlated with target-P3 latencies (*r* = 0.56, *p* < 0.001). Error rates for children in the reward conditions positively correlated with target-P2 latencies (high monetary reward: *r* = 0.59, *p* < 0.001; low monetary reward: *r* = 0.51, *p* < 0.005; high social reward: *r* = 0.618, *p* < 0.001; low social reward: *r* = 0.54, *p* < 0.002). For adults, RTs in monetary reward conditions negatively correlated with target-P3 amplitude (high reward: *r* = −0.60, *p* < 0.001; low reward: *r* = −0.61, *p* < 0.001), and RTs in low social condition positively correlated with target-P3 latencies (*r* = 0.58, *p* < 0.001).

## Discussion

The current study explored the neurodevelopment of social and monetary reward processes in adolescents compared with that in children and adults. The behavioral performances of individuals increased with age. Children and adolescents had stronger motivation (larger cue-P3) for rewards and devoted stronger attentional control (larger target-P3) than adults. Adolescents were more sensitive to social rewards than children and adults, and children had stronger emotional sensitivity for monetary rewards than for social rewards. The current findings offer the clinical implications that social rewards could be used to motivate adolescents’ behaviors, and that monetary rewards might be more effective than social rewards for children and adults.

### The Development of Reward Processes in Children, Adolescents and Adults

Currently, developmental differences in response speed and performance accuracy among three age groups have been found; adolescents and adults have faster response speeds than children, which might reveal that children’s ability to utilize reward information and display proper behavior to obtain a reward is less developed (Crone and van der Molen, 2004; Cassotti et al., 2011). Children and adolescents make more erroneous responses than adults, which is consistent with previous developmental findings on reward processes (Wang et al., 2017). More importantly, children and adolescents have larger cue-P3 amplitudes than adults. This finding suggests that children and adolescents might show stronger motivation to reward-related cue information and devote more neural effort compared to adults.

Target-P2 reflects an individual’s emotional sensitivity to reward stimuli (Doñamayor et al., 2012). The current findings show that children and adolescents have larger target-P2 amplitudes than adults, and adolescents have shorter target-P2 latencies than children. These findings indicate that rewards could lead to stronger emotional responses in children and adolescents than in adults, and adolescents have faster emotional reactivity to rewards than children. This might be due to the following factors. First, the emotion regulation model indicates that the amygdala, ventral striatum and ventromedial PFC (vmPFC) play key roles in generating emotion (Ochsner et al., 2012). Adolescents have stronger activation in the ventral striatum and amygdala in response to reward and emotional stimuli than adults (Guyer et al., 2008; Somerville and Casey, 2010). Hence, processing reward stimuli could trigger stronger emotional responses in adolescents, which was reflected by more positive target-P2 amplitudes. Second, adolescence is a period when pubertal hormone secretion, such as testosterone and estradiol, increases (Forbes et al., 2010; Op de Macks et al., 2011; Galvan, 2013). It has been reported that increased levels of hormones make adolescents more emotionally expressive (Nottelmann et al., 1987; Marceau et al., 2013). Beyond these findings, it is known that a social reward is a pleasant feeling of human interaction (Kohls et al., 2009; Stavropoulos and Carver, 2013; Cox et al., 2015). Target-P3 relates to the attentional capture of reward and reward-related task accomplishments (Broyd et al., 2012; Flores et al., 2015; Wei et al., 2015). Both monetary and social rewards were more effective in triggering attentional capture in children and adolescents than in adults. Similarly, the current study indicated that a higher reward magnitude was associated with a more positive target-P3 amplitudes in adolescents and adults. Adolescents and adults were more sensitive to reward magnitudes, and different reward magnitudes could affect their target execution differently. For children, target-P3 amplitudes were larger only in the high reward magnitude condition compared to the low and non-reward conditions. These findings confirmed that the ability of children to use reward information to influence attention is not fully developed (Crone and van der Molen, 2004; Cassotti et al., 2011). Taken together, the current results of target-P3 suggested that reward magnitudes could modulate attentional control in adolescents and adults more precisely than in children (Steinberg, 2008; Van Leijenhorst et al., 2009).

Similar to previous studies, children exhibited larger FRN responses for rewards than adolescents and adults (Eppinger et al., 2009; Hämmerer et al., 2011; Crowley et al., 2013). In addition, for children and adolescents, there were differences between the reward feedback and non-reward feedback regarding FRN amplitudes. However, the comparison analyses showed that there were no differences between reward and non-reward feedback in the adult group. Contrary to previous research, reward feedback elicited more negative FRN than non-reward feedback (Eppinger et al., 2009; Hämmerer et al., 2011; Crowley et al., 2013). This was mainly due to the differences in the experimental design. Previous monetary reward tasks were probabilistic learning, and non-reward or punishment feedback was more unexpected; hence, it elicited more negative FRN. In the current study, the type of feedback could be expected based on the information of the cue and target response. When participants were expecting feedback, compared with non-reward trials, the response correctness and response speed needed to be evaluated at the same time in reward-related trials. Therefore, reward feedback was more unexpected than neutral feedback, and the FRN for reward feedback had larger negative values.

The correlation analyses between behavioral data and ERP data also showed interesting age-related findings. Behavioral performances of adolescents only correlated with their neural responses under the social reward condition; however, for children and adults, a significant association between behavioral performances and neural responses was found in both social and monetary conditions. These findings suggest that adolescents’ reward processes were mainly sensitive to social rewards, and their behavioral performances for monetary rewards were not strictly related to their neural activities. Furthermore, adolescents’ reward-related behavioral performance is related to neural responses of the cognitive control process (target-P3), which might reveal that cognitive control processes are essential for adolescents’ social reward behaviors. These results were in agreement with the adult findings in the present study. This might illustrate that with individual development, adolescents have reached reward process patterns similar to those of adults. For children, the behavioral performances of reward processes were associated with emotional responses to reward cue information, which could be induced by reward processes in children being affected by their emotion processes.

### The Comparisons Between Social and Monetary Reward Processes

Another focus of the current study was the motivation differences toward monetary and social rewards among children, adolescents and adults. The performance speed for all age groups was accelerated after high and low reward cues compared to after non-reward cues, and high reward cues induced larger cue-P3 responses than low and non-reward cues. These findings revealed that both monetary and social rewards were attractive and that the setting of the reward magnitudes was valid, which might further indicate that rewards induce an individual’s motivation and encourage them to perform faster (Kohls et al., 2009, 2011; Spreckelmeyer et al., 2009; Demurie et al., 2011, 2012; Stavropoulos and Carver, 2013). Generally, the current results for adults are in agreement with previous studies showing a stronger propensity of monetary conditions to elicit neural reward responses, better accuracy and faster RTs compared to social reward conditions (Barman et al., 2015).

Children and adolescents subjectively rated higher motivation toward social rewards than monetary rewards, and adults rated their motivation toward the two reward types equally, which was in agreement with prior studies (Wang et al., 2017). Moreover, cue-P3 was related to the motivation elicited by reward cues (Nieuwenhuis et al., 2005; Goldstein et al., 2006; Kohls et al., 2011; Broyd et al., 2012; Cox et al., 2015; Flores et al., 2015; Wei et al., 2015), but there were no reward type-related differences in cue-P3 responses, which indicated that there were no differences in the neural processing of cue-induced motivation on monetary rewards and social rewards. Therefore, when measuring an individual’s motivation for a reward, objective performance and subjective ratings should be simultaneously considered because they reflect different aspects of individual motivation.

The current study found that children had larger target-P2 responses to monetary rewards than to social rewards only in the low reward condition, which indicated that children were more emotionally sensitive to monetary rewards compared to social rewards with a medium level of motivation. It was also currently observed that adolescents showed shorter FRN latencies to social rewards than monetary rewards in the low reward condition. This finding might indicate that with a medium level of motivation, adolescents had faster neural processes for social feedback than monetary feedback, and it further illustrates adolescent’s hypersensitivity to social rewards. Children showed shorter FRN latencies to monetary feedback than to social feedback with a high level of motivation, which might reveal that children had faster neural processing speeds regarding monetary feedback compared to social feedback. Taken together, these findings might be because children aged 9–10 years old have established a comparably mature concept of money and an extremely strong desire to own and to use money (Grunberg and Anthony, 1980; Berti and Bombi, 1981).

Interestingly, all age groups showed more positive target-P3 responses for monetary rewards compared to social rewards; this finding might demonstrate that monetary rewards induced larger attentional resources and allocation to accomplish the task compared to social rewards. In contrast to the fixed probability of obtaining rewards in previous studies (Kohls et al., 2011; Cox et al., 2015; Flores et al., 2015), the final obtained reward depended entirely on the participants’ performances during the target response phase in the current study. Furthermore, more attention resources are allocated to targets when individuals have a stronger motivation for a certain reward (Baines et al., 2011; Kaltwasser et al., 2013; Wei et al., 2015), and all participants showed a more positive target-P3 for monetary rewards compared to social rewards in the current study. Taken together, the target-P3 in incentive delay tasks was sensitive to both reward type and reward magnitude if the target duration was fixed, and there was a close relationship between the performance of the participants and the final obtained reward.

There were several limitations in the current study. First, the current design was cross-sectional, which might induce more noise and variance in the developmental analysis. In the future, more age groups (such as a 9–11-year-old group and/or a-15–18-year-old group) should be recruited to explore a more elaborate developmental trajectory of reward processes. The measurement on the individual traits (personality, impulsivity, risk-taking etc.) should also be considered in the future study to enhance the inference for psychological processes. Second, social rewards are culturally shaped, and the current study only enrolled Asian participants. One should be careful when applying the current findings to Western culture. Third, the comparison between feedback processes required further refinement since the current feedback stimuli were perceptually different (shiny coins vs. complex faces). This unbalanced design might lead to differences in waveforms that are not due to reward magnitude effects. Future studies should require participants to rate the reward level of the feedback images and then use these subjective ratings as a covariate for analysis to reduce the effects of an unequal stimulus design.

## Conclusion

Both monetary and social rewards may be incentives for increasing motivation and facilitating behaviors. Children and adolescents had higher motivation for rewards and devoted more neural effort to execute attentional control than adults. Adolescents showed larger emotional responses to rewards compared with children and adults. Compared to social rewards, monetary rewards could induce stronger emotional reactivity in children. Individuals were better able to produce a high level of neural effort for attentional control for monetary rewards than for social rewards. The current study sheds light on the neurodevelopment of reward processes and the influence of various reward types on development.

## Data Availability Statement

All datasets generated for this study are included in the article/supplementary material.

## Ethics Statement

The studies involving human participants were reviewed and approved by the Institute of Psychology, Chinese Academy of Sciences. Written informed consent to participate in this study was provided by the participants’ legal guardian/next of kin.

## Author Contributions

DW: conceptualization, methodology, software, validation, formal analysis, investigation, data curation, and writing – original draft. TL: conceptualization, methodology, software, validation, formal analysis, investigation, data curation, writing – review and editing, supervision, project administration, and funding acquisition. JS: conceptualization, supervision, and project administration.

## Conflict of Interest

The authors declare that the research was conducted in the absence of any commercial or financial relationships that could be construed as a potential conflict of interest.

## References

[B1] Albert D. Chein J. Steinberg L. (2013). The teenage brain: peer influences on adolescent decision making. *Curr. Dir. Psychol. Sci.* 22 114–120. 10.1177/0963721412471347 25544805 PMC4276317

[B2] Altikulaç S. Bos M. G. N. Foulkes L. Crone E. A. van Hoorn J. (2019). Age and gender effects in sensitivity to social rewards in adolescents and young adults. *Front. Behav. Neurosci.* 13:171. 10.3389/fnbeh.2019.00171 31417377 PMC6681770

[B3] Anderson B. A. (2016). Social reward shapes attentional biases. *Cogn. Neurosci.* 7 30–36. 10.1080/17588928.2015.1047823 25941868 PMC4654995

[B4] Baines S. Ruz M. Rao A. Denison R. Nobre A. C. (2011). Modulation of neural activity by motivational and spatial biases. *Neuropsychologia* 49 2489–2497. 10.1016/j.neuropsychologia.2011.04.029 21570417

[B5] Bar-Haim Y. Fox N. A. Benson B. Guyer A. E. Williams A. Nelson E. E. (2009). Neural correlates of reward processing in adolescents with a history of inhibited temperament. *Psychol. Sci.* 20 1009–1018. 10.1111/j.1467-9280.2009.02401.x 19594857 PMC2785902

[B6] Barman A. Richter S. Soch J. Deibele A. Richter A. Assmann A. (2015). Gender-specific modulation of neural mechanisms underlying social reward processing by Autism Quotient. *Soc. Cogn. Affect. Neurosci.* 10 1537–1547. 10.1093/scan/nsv044 25944965 PMC4631150

[B7] Beck S. M. Locke H. S. Savine A. C. Jimura K. Braver T. S. (2010). Primary and secondary rewards differentially modulate neural activity dynamics during working memory. *PLoS One* 5:e9251. 10.1371/journal.pone.0009251 20169080 PMC2821928

[B8] Berti A. E. Bombi A. S. (1981). The development of the concept of money and its value: a longitudinal study. *Child Dev.* 52 1179–1182. 10.2307/1129504

[B9] Bhanji J. P. Delgado M. R. (2014). The social brain and reward: social information processing in the human striatum. *Wiley Interdiscip. Rev. Cogn. Sci.* 5 61–73. 10.1002/wcs.1266 24436728 PMC3890330

[B10] Bjork J. M. Knutson B. Fong G. W. Caggiano D. M. Bennett S. M. Hommer D. W. (2004). Incentive-elicited brain activation in adolescents: similarities and differences from young adults. *J. Neurosci.* 24 1793–1802. 10.1523/JNEUROSCI.4862-03.2004 14985419 PMC6730402

[B11] Bjork J. M. Smith A. R. Chen G. Hommer D. W. (2010). Adolescents, adults and rewards: comparing motivational neurocircuitry recruitment using fMRI. *PLoS One* 5:e11440. 10.1371/journal.pone.0011440 20625430 PMC2897849

[B12] Blakemore S.-J. (2008). The social brain in adolescence. *Nat. Rev. Neurosci.* 9 267–277. 10.1038/nrn2353 18354399

[B13] Blakemore S.-J. Choudhury S. (2006). Development of the adolescent brain: implications for executive function and social cognition. *J. Child. Psychol. Psychiatry* 47 296–312. 10.1111/j.1469-7610.2006.01611.x 16492261

[B14] Blakemore S.-J. Mills K. L. (2014). Is adolescence a sensitive period for sociocultural processing? *Annu. Rev. Psychol.* 65 187–207. 10.1146/annurev-psych-010213-115202 24016274

[B15] Blakemore S.-J. Robbins T. W. (2012). Decision-making in the adolescent brain. *Nat. Neurosci.* 15 1184–1191. 10.1038/nn.3177 22929913

[B16] Braams B. R. van Duijvenvoorde A. C. Peper J. S. Crone E. A. (2015). Longitudinal changes in adolescent risk-taking: a comprehensive study of neural responses to rewards, pubertal development, and risk-taking behavior. *J. Neurosci.* 35 7226–7238. 10.1523/JNEUROSCI.4764-14.2015 25948271 PMC6605271

[B17] Bress J. N. Meyer A. Proudfit G. H. (2015). The stability of the feedback negativity and its relationship with depression during childhood and adolescence. *Dev. Psychopathol.* 27 1285–1294. 10.1017/S0954579414001400 26439074

[B18] Broyd S. J. Richards H. J. Helps S. K. Chronaki G. Bamford S. Sonuga-Barke E. J. S. (2012). An electrophysiological monetary incentive delay (e-MID) task: a way to decompose the different components of neural response to positive and negative monetary reinforcement. *J. Neurosci. Methods* 209 40–49. 10.1016/j.jneumeth.2012.05.015 22659003

[B19] Burnett S. Sebastian C. Kadosh K. C. Blakemore S. J. (2011). The social brain in adolescence: evidence from functional magnetic resonance imaging and behavioural studies. *Neurosci. Biobehav. Rev.* 35 1654–1664. 10.1016/j.neubiorev.2010.10.011 21036192 PMC4538788

[B20] Cao Z. Bennett M. Orr C. Icke I. Banaschewski T. Barker G. J. (2018). Mapping adolescent reward anticipation, receipt, and prediction error during the monetary incentive delay task. *Hum. Brain Mapp.* 40 262–283. 10.1002/hbm.24370 30240509 PMC6865381

[B21] Casey B. Jones R. M. Somerville L. H. (2011). Braking and accelerating of the adolescent brain. *J. Res. Adolesc.* 21 21–33. 10.1111/j.1532-7795.2010.00712.x 21475613 PMC3070306

[B22] Casey B. J. Jones R. M. Hare T. A. (2008). The adolescent brain. *Ann. N. Y. Acad. Sci.* 1124 111–126. 10.1196/annals.1440.010 18400927 PMC2475802

[B23] Cassotti M. Houdé O. Moutier S. (2011). Developmental changes of win-stay and loss-shift strategies in decision making. *Child Neuropsychol.* 17 400–411. 10.1080/09297049.2010.547463 21390919

[B24] Chan R. C. Li Z. Li K. Zeng Y.-W. Xie W.-Z. Yan C. (2016). Distinct processing of social and monetary rewards in late adolescents with trait anhedonia. *Neuropsychology* 30 274–280. 10.1037/neu0000233 26280299

[B25] Cohen M. X. Young J. Baek J.-M. Kessler C. Ranganath C. (2005). Individual differences in extraversion and dopamine genetics predict neural reward responses. *Cogn. Brain Res.* 25 851–861. 10.1016/j.cogbrainres.2005.09.018 16289773

[B26] Cox A. Kohls G. Naples A. J. Mukerji C. E. Coffman M. C. Rutherford H. J. (2015). Diminished social reward anticipation in the broad autism phenotype as revealed by event-related brain potentials. *Soc. Cogn. Affect. Neurosci.* 10 1357–1364. 10.1093/scan/nsv024 25752905 PMC4590535

[B27] Crone E. A. Dahl R. E. (2012). Understanding adolescence as a period of social-affective engagement and goal flexibility. *Nat. Rev. Neurosci.* 13 636–650. 10.1038/nrn3313 22903221

[B28] Crone E. A. van der Molen M. W. (2004). Developmental changes in real life decision making: performance on a gambling task previously shown to depend on the ventromedial prefrontal cortex. *Dev. Neuropsychol.* 25 251–279. 10.1207/s15326942dn2503_2 15147999

[B29] Crowley M. J. Wu J. Hommer R. E. South M. Moflese P. J. Fearon R. M. P. (2013). A developmental study of the feedback-related negativity from 10-17 years: age and sex effects for reward versus non-reward. *Dev. Neuropsychol.* 38 595–612. 10.1080/87565641.2012.694512 24219697 PMC3992989

[B30] Delgado M. R. (2007). Reward-related responses in the human striatum. *Ann. N. Y. Acad. Sci.* 1104 70–88. 10.1196/annals.1390.002 17344522

[B31] Demurie E. Roeyers H. Baeyens D. Sonuga-Barke E. (2011). Common alterations in sensitivity to type but not amount of reward in ADHD and autism spectrum disorders. *J. Child Psychol. Psychiatry* 52 1164–1173. 10.1111/j.1469-7610.2010.02374.x 21223259

[B32] Demurie E. Roeyers H. Baeyens D. Sonuga-Barke E. (2012). The effects of monetary and social rewards on task performance in children and adolescents: liking is not enough. *Int. J. Methods Psychiatr. Res.* 21 301–310. 10.1002/mpr.1370 23148022 PMC6878378

[B33] Doñamayor N. Schoenfeld M. A. Münte T. F. (2012). Magneto- and electroencephalographic manifestations of reward anticipation and delivery. *Neuroimage* 62 17–29. 10.1016/j.neuroimage.2012.04.038 22561022

[B34] Eppinger B. Mock B. Kray J. (2009). Developmental differences in learning and error processing: evidence from ERPs. *Psychophysiology* 46 1043–1053. 10.1111/j.1469-8986.2009.00838.x 19497006

[B35] Ernst M. Nelson E. E. Jazbec S. McClure E. B. Monk C. S. Leibenluft E. (2005). Amygdala and nucleus accumbens in responses to receipt and omission of gains in adults and adolescents. *Neuroimage* 25 1279–1291. 10.1016/j.neuroimage.2004.12.038 15850746

[B36] Ernst M. Pine D. S. Hardin M. (2006). Triadic model of the neurobiology of motivated behavior in adolescence. *Psychol. Med.* 36 299–312. 10.1017/S0033291705005891 16472412 PMC2733162

[B37] Ethridge P. Kujawa A. Dirks M. A. Arfer K. B. Kessel E. M. Klein D. N. (2017). Neural responses to social and monetary reward in early adolescence and emerging adulthood. *Psychophysiology* 54 1786–1799. 10.1111/psyp.12957 28700084 PMC5757310

[B38] Ferdinand N. K. Kray J. (2014). Developmental changes in performance monitoring: how electrophysiological data can enhance our understanding of error and feedback processing in childhood and adolescence. *Behav. Brain Res.* 263 122–132. 10.1016/j.bbr.2014.01.029 24487012

[B39] Flores A. Münte T. F. Doñamayor N. (2015). Event-related EEG responses to anticipation and delivery of monetary and social reward. *Biol. Psychol.* 109 10–19. 10.1016/j.biopsycho.2015.04.005 25910956

[B40] Flores L. E. Jr. Eckstrand K. L. Silk J. S. Allen N. B. Ambrosia M. Healey K. L. (2018). Adolescents’ neural response to social reward and real-world emotional closeness and positive affect. *Cogn. Affect. Behav. Neurosci.* 18 705–717. 10.3758/s13415-018-0598-0 29943174 PMC7108758

[B41] Forbes E. E. Dahl R. E. (2012). Research review: altered reward function in adolescent depression: What, when and how? *J. Child Psychol. Psychiatry* 53 3–15. 10.1111/j.1469-7610.2011.02477.x 22117893 PMC3232324

[B42] Forbes E. E. Ryan N. D. Phillips M. L. Manuck S. B. Worthman C. M. Moyles D. L. (2010). Healthy adolescents’ neural response to reward: associations with puberty, positive affect, and depressive symptoms. *J. Am. Acad. Child Adolesc. Psychiatry* 49 162–172. 10.1097/00004583-201002000-00010 20215938 PMC2837556

[B43] Foti D. Weinberg A. Bernat E. M. Proudfit G. H. (2015). Anterior cingulate activity to monetary loss and basal ganglia activity to monetary gain uniquely contribute to the feedback negativity. *Clin. Neurophysiol.* 126 1338–1347. 10.1016/j.clinph.2014.08.025 25454338 PMC4385748

[B44] Foulkes L. Blakemore S. J. (2016). Is there heightened sensitivity to social reward in adolescence? *Curr. Opin. Neurobiol.* 40 81–85. 10.1016/j.conb.2016.06.016 27420376

[B45] Foulkes L. Neumann C. S. Roberts R. McCrory E. Viding E. (2017). Social reward questionnaire-adolescent version and its association with callousunemotional traits. *R. Soc. Open Sci.* 4:160991. 10.1098/rsos.160991 28484617 PMC5414254

[B46] Galvan A. (2010). Adolescent development of the reward system. *Front. Hum. Neurosci.* 4:6 10.3389/neuro.09.006.2010 PMC2826184 20179786

[B47] Galvan A. (2013). The teenage brain: sensitivity to rewards. *Curr. Dir. Psychol. Sci.* 22 88–93. 10.1177/0963721413480859 PMC3992953 24761055

[B48] Galvan A. Hare T. A. Parra C. E. Penn J. Voss H. Glover G. (2006). Earlier development of the accumbens relative to orbitofrontal cortex might underlie risk-taking behavior in adolescents. *J. Neurosci.* 26 6885–6892. 10.1523/JNEUROSCI.1062-06.2006 16793895 PMC6673830

[B49] Geier C. Luna B. (2009). The maturation of inventive processing and cognitive control. *Pharmacol. Biochem. Behav.* 93 212–221. 10.1016/j.pbb.2009.01.021 19593842 PMC2792989

[B50] Geier C. F. Terwilliger R. Teslovich T. Velanova K. Luna B. (2010). Immaturities in reward processing and its influence on inhibitory control in adolescence. *Cereb. Cortex* 20 1613–1629. 10.1093/cercor/bhp225 19875675 PMC2882823

[B51] Gilbert K. E. Luking K. R. Pagliaccio D. Luby J. Barch D. M. (2019). Dampening positive affect and neural reward responding in healthy children: implications for affective inflexibility. *J. Clin. Child Adolesc. Psychol.* 48 120–130. 10.1080/15374416.2016.1233502 27819484 PMC5420488

[B52] Goldstein R. Z. Cottone L. A. Jia Z. Maloney T. Volkow N. D. Squires N. K. (2006). The effect of graded monetary reward on cognitive event-related potentials and behavior in young healthy adults. *Int. J. Psychophysiol.* 62 272–279. 10.1016/j.ijpsycho.2006.05.006 16876894 PMC2424251

[B53] Gonzalez-Gadea M. L. Sigman M. Rattazzi A. Lavin C. Rivera-Rei A. Marino J. (2016). Neural markers of social and monetary rewards in children with Attention-Deficit/Hyperactivity Disorder and Autism Spectrum Disorder. *Sci. Rep.* 6:30588. 10.1038/srep30588 27464551 PMC4964357

[B54] Grunberg N. E. Anthony B. J. (1980). Monetary awareness in children. *Basic Appl. Soc. Psychol.* 1 343–350. 10.1207/s15324834basp0104_5

[B55] Guyer A. E. Choate V. R. Pine D. S. Nelson E. E. (2012). Neural circuitry underlying affective response to peer feedback in adolescence. *Soc. Cogn. Affect. Neurosci.* 7 81–92. 10.1093/scan/nsr043 21828112 PMC3252630

[B56] Guyer A. E. Monk C. S. Mcclure-Tone E. B. Nelson E. E. Roberson-Nay R. Adler A. D. (2008). A developmental examination of amygdala response to facial expressions. *J. Cogn. Neurosci.* 20 1565–1582. 10.1162/jocn.2008.20114 18345988 PMC2902865

[B57] Guyer A. E. Nelson E. E. Perez-Edgar K. Hardin M. G. Roberson-Nay R. Monk C. S. (2006). Striatal functional alteration in adolescents characterized by early childhood behavioral inhibition. *J. Neurosci.* 26 6399–6405. 10.1523/JNEUROSCI.0666-06.2006 16775126 PMC6674047

[B58] Hämmerer D. Li S.-C. Müller V. Lindenberger U. (2011). Life span differences in eletrophysiological correlates of monitoring gains and losses during probabilistic reinforcement learning. *J. Cogn. Neurosci.* 23 579–592. 10.1162/jocn.2010.21475 20377358

[B59] Heldmann M. Russeler J. Munte T. F. (2008). Internal and external information in error processing. *BMC Neurosci.* 9:33. 10.1186/1471-2202-9-33 18366727 PMC2291472

[B60] Helfinstein S. M. Benson B. Perez-Edgar K. Bar-Haim Y. Detloff A. Pine D. S. (2011). Striatal responses to negative monetary outcomes differ between temperamentally inhibited and non-inhibited adolescents. *Neuropsychologia* 49 479–485. 10.1016/j.neuropsychologia.2010.12.015 21167189 PMC3065071

[B61] Holroyd C. B. Coles M. G. (2002). The neural basis of human error processing: reinforcement learning, dopamine, and the error-related negativity. *Psychol. Rev.* 109 679–709. 10.1037/0033-295X.109.4.679 12374324

[B62] Holroyd C. B. Nieuwenhuis S. Yeung N. Cohen J. D. (2003). Errors in reward prediction are reflected in the event-related brain potential. *Neuroreport* 14 2481–2484. 10.1097/00001756-200312190-00037 14663214

[B63] Hoogendam J. M. Kahn R. S. Hillegers M. H. J. van Buuren M. Vink M. (2013). Different developmental trajectories for anticipation and receipt of reward during adolescence. *Dev. Cogn. Neurosci.* 6 113–124. 10.1016/j.dcn.2013.08.004 24055865 PMC6987765

[B64] Izuma K. Saito D. N. Sadato N. (2008). Processing of social and monetary rewards in the human striatum. *Neuron* 58 284–294. 10.1016/j.neuron.2008.03.020 18439412

[B65] Jia S. Zhang W. Li P. Feng T. Li H. (2013). Attitude toward money modulates outcome processing: an ERP study. *Soc. Neurosci.* 8 43–51. 10.1080/17470919.2012.713316 22856426

[B66] Kaltwasser L. Ries S. Sommer W. Knight R. T. Willems R. M. (2013). Independence of valence and reward in emotional word processing: electrophysiological evidence. *Front. Psychol.* 4:168. 10.3389/fpsyg.2013.00168 23580258 PMC3619106

[B67] Kennis M. Rademaker A. R. Geuze E. (2013). Neural correlates of personality: an integrative review. *Neurosci. Biobehav. Rev.* 37 73–95. 10.1016/j.neubiorev.2012.10.012 23142157

[B68] Knutson B. Bhanji J. P. Cooney R. E. Atlas L. Y. Gotlib I. H. (2008). Neural responses to monetary incentives in major depression. *Biol. Psychiatry* 63 686–692. 10.1016/j.biopsych.2007.07.023 17916330 PMC2290738

[B69] Kohls G. Peltzer J. Herpertz-Dahlmann B. Konrad K. (2009). Differential effects of social and non-social reward on response inhibition in children and adolescents. *Dev. Sci.* 12 614–625. 10.1111/j.1467-7687.2009.00816.x 19635087

[B70] Kohls G. Peltzer J. Schulte-Rüther M. Kamp-Becker I. Remschmidt H. Herpertz-Dahlmann B. (2011). Atypical brain responses to reward cues in autism as revealed by event-related potentials. *J. Autism Dev. Disord.* 41 1523–1533. 10.1007/s10803-011-1177-1 21290174

[B71] Kujawa A. Carroll A. Mumper E. Mukherjee D. Kessel E. M. Olino T. (2018). A longitudinal examination of event-related potentials sensitive to monetary reward and loss feedback from late childhood to middle adolescence. *Int. J. Psychophysiol.* 132 323–330. 10.1016/j.ijpsycho.2017.11.001 29113953 PMC5934346

[B72] Kujawa A. Proudfit G. H. Kessel E. M. Dyson M. Olino T. Klein D. N. (2015). Neural reactivity to monetary rewards and losses in childhood: longitudinal and concurrent associations with observed and self-reported positive emotionality. *Biol. Psychol.* 104 41–47. 10.1016/j.biopsycho.2014.11.008 25433097 PMC4300239

[B73] Lamm C. Benson B. E. Guyer A. (2014). Longitudinal study of striatal activation to reward and loss anticipation from mid-adolescence into late adolescence/early adulthood. *Brain Cogn.* 89 51–60. 10.1016/j.bandc.2013.12.003 24485273 PMC4113563

[B74] Leotti L. A. Delgado M. R. (2014). The value of exercising control over monetary gains and losses. *Psychol. Sci.* 25 596–604. 10.1177/0956797613514589 24390827 PMC3970926

[B75] Li Q. Wang Y. Yang Z. Dai W. Zheng Y. Sun Y. (2020). Dysfunctional cognitive control and reward processing in adolescents with Internet gaming disorder. *Pyschophysiology* 57:e13469. 10.1111/psyp.13469 31456249

[B76] Lin A. Adolphs R. Rangel A. (2012). Social and monetary reward learning engage overlapping neural substrates. *Soc. Cogn. Affect. Neurosci.* 7 274–281. 10.1093/scan/nsr006 21427193 PMC3304477

[B77] Luking K. R. (2015). *How Gains and Losses Influence the Brain and Behavior: Relations to Age, Risk for Depression, and Individual Differences.* Ph.D. thesis, Graduate School of Arts and Sciences, Cambridge, MA.

[B78] Luking K. R. Infantolino Z. P. Nelson B. D. Hajcak G. (2019). Age-typical changes in neural reward response are moderated by maternal anhedonia. *Psychophysiology* 56:e13358. 10.1111/psyp.13358 30811613

[B79] Luking K. R. Pagliaccio D. Luby J. L. Barch D. M. (2016). Reward processing and risk for depression across development. *Trends Cogn. Sci.* 20 456–468. 10.1016/j.tics.2016.04.002 27131776 PMC4875800

[B80] Marceau K. Horwitz B. N. Ganiban J. M. Reiss D. Narusyte J. Spotts E. L. (2013). Gene-environment correlation underlying the association between parental negativity and adolescent externalizing problems. *Child Dev.* 84 2031–2046. 10.1111/cdev.12094 23573986 PMC3928634

[B81] Martin C. A. Kelly T. H. Rayens M. K. Brogli B. R. Brenzel A. Smith W. J. (2002). Sensation seeking, puberty, and nicotine, alcohol, and marijuana use in adolescence. *J. Am. Acad. Child Adolesc. Psychiatry* 41 1495–1502. 10.1097/00004583-200212000-00022 12447037

[B82] McKewen M. Skippen P. Cooper P. S. Wong A. S. W. Michie P. T. Lenroot R. (2019). Does cognitive control ability mediate the relationship between reward-related mechanism, impulsivity, and maladaptive outcomes in adolescence and young adulthood? *Cogn. Affect. Behav. Neurosci.* 19 653–676. 10.3758/s13415-019-00722-2 31119652

[B83] Nees F. Vollstädt-Klein S. Fauth-Bühler M. Steiner S. Mann K. Poustka L. (2012). A target sample of adolescents and reward processing: same neural and behavioral correlates engaged in common paradigms? *Exp. Brain Res.* 223 429–439. 10.1007/s00221-012-3272-8 23108370

[B84] Nieuwenhuis S. Aston-Jones G. Cohen J. D. (2005). Decision making, the P3, and the locus coeruleus-norepinephrine system. *Psychol. Bull.* 131 510–532. 10.1037/0033-2909.131.4.510 16060800

[B85] Nottelmann E. D. Susman E. J. Inoff-Germain G. Jr. Loriaux D. L. Chrousos G. P. (1987). Developmental processes in early adolescence: relationships between adolescent adjustment problems and chronologic age, pubertal stage, and puberty-related serum hormone levels. *J. Pediatri.* 110 473–480. 3819952 10.1016/s0022-3476(87)80521-8

[B86] Ochsner K. N. Silvers J. A. Buhle J. T. (2012). Functional imaging studies of emotion regulation: a synthetic review and evolving model of the cognitive control of emotion. *Ann. N. Y. Acad. Sci.* 1251:E1. 10.1111/j.1749-6632.2012.06751.x 23025352 PMC4133790

[B87] Oldham S. Murawski C. Fornito A. Youssef G. Yücel M. Lornzetti V. (2018). The anticipation and outcome phases of reward and loss processing: a neuroimaging meta-analysis of the monetary incentive delay task. *Hum. Brain Mapp.* 39 3398–3418. 10.1002/hbm.24184 29696725 PMC6055646

[B88] Olino T. M. Silk J. S. Osterritter C. Forbes E. E. (2015). Social reward in youth at risk for depression: a preliminary investigation of subjective and neural differences. *J. Child Adolesc. Psychopharmacol.* 25 711–721. 10.1089/cap.2014.0165 26469133 PMC4653819

[B89] Op de Macks Z. A. Moor B. G. Overgaauw S. Güroðlu B. Dahl R. E. Crone E. A. (2011). Testosterone levels correspond with increased ventral striatum activation in response to monetary rewards in adolescents. *Dev. Cogn. Neurosci.* 1 506–516. 10.1016/j.dcn.2011.06.003 22436568 PMC6987540

[B90] Oumeziane B. A. Schryer-Praga J. Foti D. (2017). “Why don’t they ‘like’ me more?”: comparing the time courses of social and monetary reward processing. *Neuropsychologia* 107 48–59. 10.1016/j.neuropsychologia.2017.11.001 29104079

[B91] Pfabigan D. M. Seidel E. M. Paul K. Grahl A. Sailer U. Lanzenberger R. (2015). Context-sensitivity of the feedback-related negativity for zero-value feedback outcomes. *Biol. Psychol.* 104 184–192. 10.1016/j.biopsycho.2014.12.007 25541513

[B92] Pfabigan D. M. Seidel E. M. Sladky R. Hahn A. Paul K. Grahl A. (2014). P300 amplitude variation is related to ventral striatum BOLD response during gain and loss anticipation: an EEG and fMRI experiment. *Neuroimage* 96 12–21. 10.1016/j.neuroimage.2014.03.077 24718288 PMC4075343

[B93] Poon J. A. Niehaus C. E. Thompson J. C. Chaplin T. M. (2019). Adolescents’ pubertal development: links between testosterone, estradiol, and neural reward processing. *Horm. Behav.* 114:104504 10.1016/j.yhbeh.2019.02.015 PMC7903811 30817913

[B94] Rademacher L. Krach S. Kohls G. Irmak A. Gründer G. Spreckelmeyer K. N. (2010). Dissociation of neural networks for anticipation and consumption of monetary and social rewards. *Neuroimage* 49 3276–3285. 10.1016/j.neuroimage.2009.10.089 19913621

[B95] Richards J. M. Plate R. C. Ernst M. (2013). A systematic review of fMRI reward paradigms used in studies of adolescents vs. adults: the impact of task design and implications for understanding neurodevlopment. *Neurosci. Biobehav. Rev.* 37 976–991. 10.1016/j.neubiorev.2013.03.004 23518270 PMC3809756

[B96] Saxe R. Haushofer J. (2008). For love or money: a common neural currency for social and monetary reward. *Neuron* 58 164–165. 10.1016/j.neuron.2008.04.005 18439400

[B97] Schacht A. Sommer W. (2009). Emotions in word and face processing: early and late cortical responses. *Brain Cogn.* 69 538–550. 10.1016/j.bandc.2008.11.005 19097677

[B98] Sheffield J. Crowley M. J. Bel-Bahar T. Desatnik A. Nolte T. Fonagy P. (2015). Reward-related neural activity and adolescent antisocial behavior in a community sample. *Dev. Neuropsychol.* 40 363–378. 10.1080/87565641.2015.1101466 26491989

[B99] Shulman E. P. Smith A. R. Silva K. Icenogle G. Duell N. Chein J. (2016). The dual systems model: review, reappraisal, and reaffirmation. *Dev. Cogn. Neurosci.* 17 103–117. 10.1016/j.dcn.2015.12.010 26774291 PMC6990093

[B100] Silverman M. H. Jedd K. Luciana M. (2015). Neural networks involved in adolescent reward processing: an activation likelihood estimation meta-analysis of functional neuroimaging studies. *Neuroimage* 122 427–439. 10.1016/j.neuroimage.2015.07.083 26254587 PMC4618189

[B101] Simon J. J. Walther S. Fiebach C. J. Friederich H.-C. Stippich C. Weisbrod M. (2010). Neural reward processing is modulated by approach and avoidance-related personality traits. *Neuroimage* 49 1868–1874. 10.1016/j.neuroimage.2009.09.016 19770056

[B102] Somerville L. H. (2013). The teenage brain: sensitivity to social evaluation. *Curr. Dir. Psychol. Sci.* 22 121–127. 10.1177/0963721413476512 24761055 PMC3992953

[B103] Somerville L. H. Casey B. J. (2010). Developmental neurobiology of cognitive control and motivational systems. *Curr. Opin. Neurobiol.* 20 236–241. 10.1016/j.conb.2010.01.006 20167473 PMC3014528

[B104] Somerville L. H. Jones R. M. Casey B. J. (2010). A time of change: behavioral and neural correlates of adolescent sensitivity to appetitive and aversive environment cues. *Brain Cogn.* 72 124–133. 10.1016/j.bandc.2009.07.003 19695759 PMC2814936

[B105] Spear L. P. (2011). Rewards, aversions and affect in adolescence: emerging convergences across laboratory animal and human data. *Dev. Cogn. Neurosci.* 1 392–400. 10.1016/j.dcn.2011.08.001 21918675 PMC3170768

[B106] Spreckelmeyer K. N. Krach S. Kohls G. Rademacher L. Irmak A. Konrad K. (2009). Anticipation of monetary and social reward differently activates mesolimbic brain structures in men and women. *Soc. Cogn. Affect. Neurosci.* 4 158–165. 10.1093/scan/nsn051 19174537 PMC2686229

[B107] Stavropoulos K. K. M. Carver L. J. (2013). Reward sensitivity to faces versus objects in children: an ERP study. *Soc. Cogn. Affect. Neurosci.* 9 1569–1575. 10.1093/scan/nst149 24036961 PMC4187274

[B108] Steinberg L. (2005). Cognitive and affetive development in adolescence. *Trends Cogn. Sci.* 9 69–74. 15668099 10.1016/j.tics.2004.12.005

[B109] Steinberg L. (2008). A social neuroscience perspective on adolescent risk-taking. *Dev. Rev.* 28 78–106. 10.1016/j.dr.2007.08.002 18509515 PMC2396566

[B110] Steinberg L. Icenogle G. Shulman E. P. Breiner K. Chein J. Bacchini D. (2017). Around the world, adolescence is a time of heightened sensation seeking and immature self-regulation. *Dev. Sci.* 21:e12532. 10.1111/desc.12532 28150391 PMC6473801

[B111] Trezza V. Damsteegt R. Achterberg E. M. Vanderschuren L. J. (2011). Nucleus accumbens μ-opioid receptors mediate social reward. *J. Neurosci.* 31 6362–6370. 10.1523/JNEUROSCI.5492-10.2011 21525276 PMC3098965

[B112] Urošević S. Collins P. Muetzel R. Lim K. Luciana M. (2012). Longitudinal changes in behavioral approach system sensitivity and brain structures involved in reward procesing during adolescence. *Dev. Psychol.* 48 1488–1500. 10.1037/a0027502 22390662 PMC3370133

[B113] van Hoorn J. Shablack H. Lindquist K. Telzer E. H. (2019). Incorporating the social context into neurocognitive models of adolescent decision-making: a neuroimaging meta-analysis. *Neurosci. Biobehav. Rev.* 101 129–142. 10.1016/j.neubiorev.2018.12.024 31006540 PMC6659412

[B114] Van Leijenhorst L. Gunther Moor B. Op de Macks Z. A. Rombouts S. A. Westenberg P. M. Crone E. A. (2010). Adolescent risky decision-making: neurocognitive development of reward and control regions. *Neuroimage* 51 345–355. 10.1016/j.neuroimage.2010.02.038 20188198

[B115] Van Leijenhorst L. Zanolie K. Van Meel C. S. Westenberg P. M. Rombouts S. A. Crone E. A. (2009). What motivates the adolescent? brain regions mediating reward sensitivity across adolescence. *Cereb. Cortex* 20 61–69. 10.1093/cercor/bhp078 19406906

[B116] Wahlstrom D. White T. Luciana M. (2010). Neurobehavioral evidence for changes in dopamine system activity during adolescence. *Neurosci. Biobehav. Rev.* 34 631–648. 10.1016/j.neubiorev.2009.12.007 20026110 PMC2845533

[B117] Wang D. Liu T. Shi J. (2017). Development of monetary and social reward processes. *Sci. Rep.* 7:11128. 10.1038/s41598-017-11558-6 28894231 PMC5594021

[B118] Wei P. Wang D. Ji L. (2015). Reward expectation regulates brain responses to task-relevant and task-irrelevant emotional words: ERP evidence. *Soc. Cogn. Affect. Neurosci.* 11 191–203. 10.1093/scan/nsv097 26245838 PMC4733331

[B119] Weigard A. Chein J. Albert D. Smith A. Steinberg L. (2014). Effects of anonymous peer observation on adolescents’ preference for immediate rewards. *Dev. Sci.* 17 71–78. 10.1111/desc.12099 24341973 PMC3869036

[B120] Weinberg A. Riesel A. Proudfit G. H. (2014). Show me the money: the impact of actual rewards and losses on the feedback negativity. *Brain Cogn.* 87 134–139. 10.1016/j.bandc.2014.03.01 24735733

[B121] Zink C. F. Tong Y. Chen Q. Bassett D. S. Stein J. L. Meyer-Lindenberg A. (2008). Know your place: neural processing of social hierarchy in humans. *Neuron* 58 273–283. 10.1016/j.neuron.2008.01.025 18439411 PMC2430590

